# Factors associated with malaria care seeking among children under 5 years of age in Mozambique: a secondary analysis of the 2018 Malaria Indicator Survey

**DOI:** 10.1186/s12936-022-04128-3

**Published:** 2022-03-24

**Authors:** Annette Cassy, Sérgio Chicumbe, Abuchahama Saifodine, Rose Zulliger

**Affiliations:** 1 Instituto Nacional de Saúde, Maputo, Mozambique; 2 Observatório Nacional de Saúde, Maputo, Mozambique; 3 U.S. President’s Malaria Initiative, USAID, Maputo, Mozambique; 4 U.S. President’s Malaria Initiative, USAID, Washington, DC USA

## Abstract

**Background:**

Mozambique is ranked fourth in a list of the 29 countries that accounted for 95% of all malaria cases globally in 2019. The aim of this study was to identify factors associated with care seeking for fever, to determine the association between knowledge about malaria and care seeking and to describe the main reasons for not seeking care among children under five years of age in Mozambique.

**Methods:**

This is a quantitative, observational study based on a secondary data analysis of the 2018 Malaria Indicator Survey. This weighted analysis was based on data reported by surveyed mothers or caregivers of children aged 0–59 months who had fever in the two weeks prior to the survey.

**Results:**

Care was reportedly sought for 69.1% [95% CI 63.5–74.2] of children aged 0–59 months old with fever. Care-seeking was significantly higher among younger children, < 6 months old (AOR = 2.47 [95% CI 1.14–5.31]), 6–11 months old (AOR = 1.75 [95% CI 1.01–3.04]) and 12–23 months old (AOR = 1.85 [95% CI 1.19–2.89]), as compared with older children (48–59 months old). In adjusted analysis, mothers from the middle (AOR = 1.66 [95% CI 0.18–3.37]) and richest (AOR = 3.46 [95% CI 1.26–9.49]) wealth quintiles were more likely to report having sought care for their febrile children than mothers from the poorest wealth quintile. Additionally, mothers with secondary or higher education level were more likely to seek care (AOR = 2.16 [95% CI 1.19–3.93]) than mothers with no education. There was no association between maternal malaria knowledge or reported exposure to malaria messages and care-seeking behaviours. The main reasons reported for not seeking care included distance to health facility (46.3% of respondents), the perception that the fever was not severe (22.4%) and the perception that treatment was not available at the health facility (15%).

**Conclusion:**

Health facility access and socioeconomic barriers continue to be important constraints to malaria service utilization in Mozambique.

## Background

Malaria remains a major public health problem in Mozambique. The country is ranked fourth in a list of the 29 countries that accounted for 95% of all malaria cases globally in 2019. Although the number of reported malaria deaths has reduced in the past decade, the country is among the six countries that accounted for more than half of the total deaths globally in 2019 [1].

The Mozambican National Malaria Control Programme (NMCP) implements social and behaviour change (SBC) interventions to ensure that by 2022 at least 70% of people seek adequate care [2]. While lower than global targets, this care-seeking target was based on national data that showed that only 63% of mothers reported care-seeking for their febrile children in 2015 [3]. According to the 2018 Malaria Indicator Survey (MIS) in Mozambique, progress was made and approximately 69% percent of mothers reported that they sought care for their febrile children [4]. However, the proportion of mothers who sought care for their febrile children in 2018 still varied by province, from 51% percent in Nampula to 88% percent in Maputo City. Prior research in Mozambique found that care-seeking for malaria was associated with higher maternal education and with lower wealth quintile [5].

The objective of this study was to identify factors associated with care-seeking for fever. As a part of that analysis, this analysis specifically sought to determine the association between knowledge about malaria and care-seeking and to describe the main reasons for not seeking care among children under 5 years of age in Mozambique using data from the 2018 MIS.

## Methods

### Study design and data source

This is a quantitative, observational study based on a secondary data analysis of the 2018 MIS data. The 2018 MIS collected nationally and provincially representative data from a representative sample of respondents. Consistent with standard MIS methodology, the sampling design had two steps: first selection of a total of 224 enumeration areas (EA) was done for urban and rural areas of each of the eleven provinces using probability proportionate to size, after which 28 households were systematically selected from each included EA. All women aged 15–49 years old who regularly resided or stayed the prior night in included households were included [6]. The survey included a total of 6,196 households and 6184 women aged 15–49 years old. The response rate for the household questionnaire was 99% percent and for the women questionnaire was 98% percent [4]. Data collection took place from March to June 2018.

### Setting

Mozambique is located on the east coast of southern Africa and is divided in 11 provinces, including the country’s capital, Maputo City. Mozambique has a surface of approximately 799,380 km^2^ [2] and an estimated population of approximately 31 million inhabitants [7]. The two most populous provinces are Nampula and Zambézia, with 6.3 million and 5.7 million inhabitants, respectively. The climate in Mozambique is tropical. The rainy season spans from October to March [2]. There is year-round transmission of malaria with seasonal peaks during the rainy season.

### Eligibility criteria

This analysis was based on data reported by surveyed mothers or caregivers about their children aged 0–59 months who had fever in the two weeks prior to the survey.

### Measures

The main outcome of this study is care-seeking of children under 5 years who had fever in the two weeks prior to data collection, as reported by mothers/guardians. In this study, care-seeking is defined as a caregiver reporting that he/she sought treatment or counselling for children under 5 years of age with fever, regardless of source of care sought [4]. Potential covariates were identified for inclusion in a predictive model based on variables identified during a literature review of “care seeking” and “treatment seeking” for fever and malaria. A total of 13 socioeconomic and demographic covariates previously shown to be associated with care-seeking [8–12] were identified and used from the 2018 MIS dataset. The covariates included child’s age, sex, place of residence (urban or rural), geographic region (province), household wealth quintile, mother’s level of education, mother’s age, child’s use of a bed net, mothers reporting hearing or seeing a message about malaria in the past 6 months, maternal comprehensive malaria knowledge and three specific questions about malaria knowledge. The following categories were considered for mother’s level of education: no education, primary education, and secondary education or higher. The mother’s level of knowledge was assessed using a composite score based on the following five variables: (i) the mother indicated fever as a symptom of malaria; (ii) the mother indicated mosquito bite as a form of malaria transmission; (iii) The mother knows that should sleep inside a mosquito net to prevent malaria; (iv) The mother knows that malaria has a cure; and (v) The mother indicated correctly at least one medicine to treat malaria.

### Statistical analysis

Descriptive statistics were used to summarize socio-economic and demographic characteristics of participants, using the children (KR) dataset. Special (svy) survey commands were used to account for the complex multilevel survey design. Data were weighted using the KR weights (wt = v005/1000000) to account for the differential selection probabilities at the EA, household, and individual levels so that any results with the regional weight factored into it would be representative at the national and regional level. Only weighted survey data are presented in this manuscript. Complex sampling logistic regression model was used to identify factors associated with care-seeking behaviour, with estimated adjusted odds ratio (AOR) and respective 95% confidence intervals (CI). All statistical analysis were performed using STATA, version 15 (Stata Corporation, College Station, Texas).

## Results

As shown in Table 1, a total of 1473 children under five years of age with a history of fever was included in the study. Care was sought for 69.1% [95% CI 63.5–74.2] of these children. Care-seeking was higher in urban areas (74.5% than in rural areas (67.3%). Care-seeking was highest in Maputo City (88.4%) and lowest in Nampula Province (52.0%).

**Table 1:** Socioeconomic and demographic characteristics of caregivers and children with fever by care seeking status in Mozambique, 2018

Characteristics	n	% of children for whom care was not sought	95% CI	% of children for whom care was sought	95% CI
Child’s age in months					
< 6	109	22.2	(13.1–34.9)	77.8	(65.1–86.9)
6–11	198	25.6	(17.7–35.5)	74.4	(64.5–82.3)
12–23	396	25.0	(18.7–32.5)	75.0	(67.5–81.3)
24–35	315	34.2	(26.0–43.4)	65.8	(56.6–74.0)
36–47	232	36.8	(27.8–46.9)	63.2	(53.1–72.2)
48–59	223	39.8	(31.2–49.1)	60.2	(50.9–68.8)
Sex of the child					
Male	740	31.8	(26.1–38.1)	68.2	(61.9–73.9)
Female	734	30.0	(23.3–37.6)	70.0	(62.4–76.7)
Place of residence					
Urban	357	25.5	(16.6–37.1)	74.5	(62.9–83.4)
Rural	1116	32.7	(326.6–39.4)	67.3	(60.6–73.4)
Region					
Niassa	102	33.9	(25.3–43.7)	66.1	(56.3–74.7)
Cabo Delgado	96	35.7	(19.2–56.5)	64.3	(43.5–80.8)
Nampula	351	48.0	(35.2–61.0)	52.0	(39.0–64.8)
Zambezia	392	27.2	(16.7–41.1)	72.8	(58.9–83.3)
Tete	135	19.3	(12.2–29.3)	80.7	(70.7–87.3)
Manica	77	18.7	(10.7–30.7)	81.3	(69.3–89.3)
Sofala	116	26.2	(13.0–45.7)	73.8	(54.3–87.0)
Inhambane	65	15.4	(7.8–28.3)	84.6	(71.7–92.2)
Gaza	55	20.6	(10.7–36.0)	79.4	(64.0–89.3)
Maputo Province	50	30.1	(20.6–41.7)	69.9	(58.3–79.4)
Maputo City	33	11.6	(5.3–23.6)	88.4	(76.4–94.7)
Wealth index					
Poorest	415	41.8	(31.6–52.8)	58.2	(47.2–68.4)
Poorer	395	32.8	(23.6–43.6)	67.2	(56.4–76.4)
Middle	278	27.6	(20.0–36.8)	72.4	(63.2–80.0)
Richer	228	21.5	(13.5–32.4)	78.5	(67.6–86.5)
Richest	157	16.9	(11.4–24.4)	83.1	(75.6–88.6)
Mother's age					
15–19	201	23.2	(14.6–34.9)	76.8	(65.1–85.4)
20–24	405	27.4	(21.2–34.5)	72.6	(65.5–78.8)
25–29	319	31.7	(23.5–41.2)	68.3	(58.8–76.5)
30–34	217	31.0	(22.5–41.0)	69.0	(59.0–77.5)
35–39	159	32.3	(21.6–45.3)	67.7	(54.7–78.4)
40–44	137	47.2	(32.4–62.5)	52.8	(37.5–67.6)
45–49	34	38.6	(20.6–60.3)	61.4	(39.7–79.4)
*Mother's level of education*					
No education	336	41.8	(30.8–53.6)	58.2	(45.6–70.7)
Primary	865	31.1	(25.7–37.2)	68.9	(62.8–74.3)
Secondary and higher	272	16.8	(11.3–24.2)	83.2	(75.8–88.7)
*Malaria prevetion methods*					
Child slept under ITN*					
No	200	41.3	(29.3–54.4)	58.7	(45.6–70.7)
Yes	1273	29.3	(24.2–35.0)	70.7	(65.0–75.8)
Mom saw or heard malaria message in past 6 months*					
No	1037	32.5	(26.1–39.8)	67.5	(45.6–70.7)
Yes	436	27.1	(20.0–35.5)	72.9	(65.0–75.8)
Maternal comprehensive malaria knowledge					
No	837	27.7	(21.4–35.0)	72.3	(65.0–78.6)
Yes	637	35.2	(27.4–43.9)	64.8	(56.1–72.6)
Malaria messages seen or heard: seek fever treatment quickly (within 24 h)*					
No	1458	31.0	(25.9–36.6)	69.0	(63.4–74.1)
Yes	15	24.4	(16.4–60.3)	75.6	(39.7–93.6)
Malaria messages seen or heard: seek treatment for fever*					
No	1445	30.9	(25.8–36.5)	69.1	(63.5–74.2)
Yes	29	31.8	(13.5–58.2)	68.2	(41.8–86.5)
Malaria messages seen or heard: malaria can kill*					
No	1330	31.7	(26.4–37.6)	68.3	(62.4–73.6)
Yes	143	23.4	(15.2–34.1)	76.6	(65.9–84.8)
Total	1473	30.9	(25.8–36.5)	69.1	(63.5–74.2)

^*^Mother's report

Younger mothers (15–19 years of age) sought care for their febrile children more often than older mothers (76.8% for 15–19 years vs. 61.4% for 45–49 years). Care-seeking was higher among those families with higher socioeconomic status and among mothers with higher education. In fact, care was sought for 58.2% of the children from the poorest wealth quintile and for 83.1% of the children from the richest quintile. Similarly, mothers with secondary or higher education sough care more frequently that those with no education, 83.2% versus 58.2%.

Care-seeking was higher for children who slept under an insecticide treated net (ITN) (70.7%) than those who did not sleep under an ITN (58.7%). It was also higher for children whose mothers reported hearing or seeing malaria message in past 6 months (72.9%) than those who did not (67.5%) but was not higher for the mothers with the comprehensive malaria knowledge (65.6%) than for those without comprehensive knowledge (73.8%).

Care-seeking was also higher among mothers who reported “hearing that malaria can kill” and those who reported “hearing that they should seek treatment for fever within 24 h”, 76.6% and 75.6%, respectively.

The vast majority of care was sought in the public sector where malaria services are provided in primary and secondary health care facilities, through mobile health services and by community health workers. Of those who sought care, 91.3% went to at their local health facility, 5.2% sought from a community health worker and 0.6% attended mobile health services. Only 0.4% sought from the private sector and 1.4% from other sources such as traditional health practitioners or traditional markets (Table 2). Most of the caregivers sought care on the first (37.3%) and second day (32.2) after fever began and 14.7% sought care on the same day (Table 3).

**Table 2:** Source of care sought by children with fever reported by caregivers in Mozambique, 2018

Place first sought treatment for fever	n	%
Public sector	987	97.05
Health facility	929	91.31
Mobile Health Services	6	0.58
Community Health Worker	52	5.15
Private sector	9	0.88
Clinic	3	0.29
Private doctor	1	0.09
Pharmacy	5	0.53
Other sources	14	1.35
Traditional market	7	0.67
Traditional practitioner	7	0.68
Other	7	0.7
Total	1017	100

**Table 3:** Number of days after fever began sought advice or treatment in Mozambique, 2018

Number of days	n	%
Same day	149	14.69
1	379	37.25
2	327	32.15
3	119	11.71
4 or more	43	4.2
Total	1017	100

### Factors associated to care-seeking for fever for children under 5 years of age

As shown in Table 4, reported care-seeking for fever was significantly higher among mothers with younger children, < 6 months old (AOR = 2.47 [95% CI 1.14–5.31]), 6–11 months old (AOR = 1.75 [95% CI 1.01–3.04]) and 12–23 months old (AOR = 1.85 [95% CI 1.19–2.89]), as compared with mothers with older children (48–59 months old).

**Table 4:** Factors associated with malaria care seeking behaviour for children under five with fever in Mozambique, 2018

Variable	Bivariable analysis			Multivariable analysis		
	OR	95% CI	p-value	AOR	95% CI	p-value
Child’s age in months						
< 6	2.32	(1.09–4.92)	0.028	2.47	(1.14–5.31)	**0.022**
6–11	1.92	(1.13–3.27)	0.017	1.75	(1.01–3.04)	**0.046**
12–23	1.99	(1.35–2.91)	0.001	1.85	(1.19–2.89)	**0.007**
24–35	1.28	(0.84–1.92)	0.246	1.18	(0.74–1.88)	0.473
36–47	1.14	(0.72–1.79)	0.583	1.07	(0.69–1.68)	0.758
48–59	1		Reference	1		Reference
Sex of the child						
Male	1		Reference	1		Reference
Female	1.09	(0.76–1.57)	0.641	1.13	(0.76–1.70)	0.532
Place of residence						
Urban	1.42	(0.76–2.63)	0.268	0.86	(0.45–1.73)	0.705
Rural	1		Reference	1		Reference
Region						
Niassa	0.26	(0.10–0.66)	0.005	0.81	(0.22–2.89)	0.740
Cabo Delgado	0.24	(0.07–0.79)	0.019	0.78	(0.18–3.37)	0.733
Nampula	0.14	(0.05–0.39)	0.000	0.47	(0.13–1.68)	0.244
Zambezia	0.35	(0.12–1.01)	0.053	1.07	(0.28–4.07)	0.925
Tete	0.55	(0.20–1.51)	0.244	1.72	(0.44–6.71)	0.435
Manica	0.57	(0.20–1.68)	0.307	1.66	(0.43–6.35)	0.461
Sofala	0.37	(0.11–1.25)	0.108	1.09	(0.26–4.66)	0.902
Inhambane	0.72	(0.23–2.27)	0.574	1.56	(0.39–6.22)	0.526
Gaza	0.51	(0.16–1.60)	0.244	1.02	(0.27–3.94)	0.974
Maputo Province	0.31	(0.11–0.82)	0.019	0.37	(0.13–1.08)	0.068
Maputo City	1		Reference	1		Reference
Wealth index						
Poorest	1		Reference	1		Reference
Poorer	1.47	(0.88–2.45)	0.142	1.25	(0.76–2.05)	0.377
Middle	1.88	(1.06–3.34)	0.032	1.66	(0.18–3.37)	**0.048**
Richer	2.62	(1.26–5.46)	0.010	1.89	(0.13–1.68)	0.128
Richest	3.53	(1.87–6.69)	0.000	3.46	(1.26–9.49)	**0.016**
Mother's education						
No education	1		Reference	1		Reference
Primary	1.58	(0.95–2.64)	0.076	1.49	(0.95–2.34)	0.085
Secondary and higher	3.55	(1.96–6.43)	0.000	2.16	(1.19–3.93)	**0.012**
Mother's age in 5 year groups						
15–19	1		Reference	1		Reference
20–24	0.80	(0.49–1.32)	0.385	0.88	(0.37–1.48)	0.705
25–29	0.65	(0.31–1.37)	0.259	0.77	(0.24–1.48)	0.552
30–34	0.67	(0.32–1.40)	0.288	0.94	(0.25–1.50)	0.874
35–39	0.63	(0.34–1.17)	0.142	1.07	(0.42–2.20)	0.856
40–44	0.34	(0.15–0.74)	0.007	0.60	(0.24–1.44)	0.280
45–49	0.48	(0.17–1.34)	0.161	0.97	(0.27–3.47)	0.963
Child slept under ITN						
No	1		Reference			
Yes	1.70	(0.98–2.94)	0.059			
Saw or heard malaria message in past 6 months						
No	1		Reference			
Yes	0.13	(0.80–2.12)	0.292			
Maternal comprehensive malaria knowledge						
No	1		Reference			
Yes	0.71	(0.43–1.16)	0.169			
Malaria messages seen or heard: seek fever treatment quickly (within 24 h)						
No	1		Reference			
Yes	1.39	(0.30–6.49)	0.674			
Malaria messages seen or heard: seek treatment for fever						
No	1		Reference			
Yes	0.96	(0.32–2.84)	0.939			
Malaria messages seen or heard: malaria can kill						
No	1		Reference			
Yes	1.52	(0.94–2.48)	0.089			

Mothers from the middle (AOR = 1.66 [95% CI 0.18–3.37]) and richest (AOR = 3.46 [95% CI 1.26–9.49]) wealth quintiles were more likely to report having sought care for their febrile children than mothers from the poorest wealth quintile. Additionally, mothers with secondary or higher education levels were more likely to seek care (AOR = 2.16 [95% CI 1.19–3.93]) than mothers with no education. Care-seeking was not associated with the sex of the child, place of residence, region, maternal age, ITN use, exposure to malaria messages or maternal comprehensive malaria knowledge.

### Reasons for not seeking care for fever

Table 5, summarizes the main reasons reported by mothers for not seeking care for a child with fever. The main reasons include distance to health facility (46.3% of respondents), perception that the fever was not severe (22.4%) and the perception that treatment was not available at the health facility (15%).

**Table 5:** Reason did not seek advice or treatment in Mozambique, 2018

Reason	n	%
Health facility is very far	211	46.26
Fever was not severe	102	22.39
Treatment was not available	73	15.93
There is no transportation	40	8.80
Other	24	5.37
Too expensive	17	3.78
Too much work	12	2.62
Did not have permission	2	0.51
Total	456	

## Discussion

Mozambique has made important progress in improving utilization of health services for fever and by 2018 the NMCP target for care-seeking was nearly achieved with 69.1% of mothers reportedly seeking care for their febrile children. This 2018 MIS complements the analysis of the 2011 and 2015 national surveys conducted in Mozambique which found lower care-seeking [5]. While these results reflect important progress since 2015, it is important to note that nearly one in every four children under five years old with fever in Mozambique did not seek care. Malaria services are freely provided in public health facilities and by community health workers in Mozambique, but appropriate access clearly continues to be a challenge, particularly for specific populations. Failure to seek care can lead to malaria morbidity, mortality, and onward transmission, compromising Mozambique’s malaria control efforts. While care-seeking is largely from public health services, some care is still sought from traditional and private providers.

Different from earlier results in Mozambique [5], this analysis showed that care-seeking in 2018 was associated with having a younger child (< 6 months old, 6–11 months old and 11–23 months old), which might be associated with a mothers’ perception that younger children are more vulnerable and require appropriate care seeking, as shown in a study from Nigeria [13]. This higher care utilization for the youngest children is a positive finding given that younger children are more likely to have worse health outcomes associated to malaria.

Care-seeking in Mozambique was also associated with the family’s middle and richest wealth quintiles, despite the fact that most of the mothers sought care in the public sector (95.9%) [4] where malaria diagnosis and treatment are free. This is indicative that while there might not be costs for services, economic factors such as indirect costs like transportation can increase the economic burden to the household, potentially inhibiting care seeking, as shown in previous studies [14, 15]. A prior study in a high burden district of Mozambique found that the median household costs associated with care-seeking for uncomplicated malaria were US$ 3.46 (IQR US$ 0.07–22.41) and US$ 81.08 (IQR US$ 39.34–88.38) per severe case. This median cost of care-seeking for uncomplicated malaria was approximately 21% (US$ 3.46) of the monthly expenditure of a family in the study province [16], indicating that malaria care-seeking may still represent a catastrophic cost for many families in Mozambique. This economic burden was also described in Malawi, with high direct and indirect costs for malaria illness episode in a country where malaria treatment is free in the public sector [17]. Additionally, maternal education continues to influence care seeking.

The main reason reported by mothers for not seeking care was the long distance to the health facility. Previous studies have also found that distance to facilities was associated with delays in care-seeking and increased risk of severe malaria [18, 19]. This provides additional evidence that there remain broader socioeconomic barriers to care seeking, underscoring the need to address systemic barriers to care such as physical access to health facilities/community case management. For example, the most frequently cited reason for not seeking care was that the facility was too far. As such, there is a critical need to improve physical and economic access to health services to improve utilization. This is reinforced by the fact that more educated and wealthier mothers were more likely to report having sought care, as previously described [10].

This study found no association between key SBC intervention objectives such as maternal comprehensive malaria knowledge and care-seeking for fever. This finding is similar to some previous studies from Ethiopia [20] and Myanmar [9]. Additionally, there was no association between reported exposure to malaria messages and care seeking. Thus, in a context such as Mozambique where there is relatively high malaria knowledge, these behavioural interventions may not be as effective as structural interventions. In the current study, sex of the child, area of residence and province are no longer significantly associated with care-seeking which is different than 2015 findings [5].

The major limitation of this study is the sample size and the relatively low number of mothers that reported hearing or seeing malaria messages in the prior 6 months. The generalizability of the finding that there was no association in this setting between exposure to malaria messages and malaria knowledge and the target behaviour of care-seeking to settings with higher coverage of SBC interventions may be limited.

## Conclusion

This study of care-seeking for fever among children 6–59 months old in Mozambique documented important improvements in care access but noted continued inequity in access to care, including distance to health facilities. The analysis of factors associated with care-seeking and of reported reasons for failure to seek care demonstrated that proximity to health facility and socioeconomic barriers such as the economic cost of care-seeking continue to be important constraints on malaria service utilization in Mozambique.

## Data Availability

Requests for the data must be made to The DHS Program at https://dhsprogram.com/methodology/survey/survey-display-527.cfm.
